# Prevalence and correlates of food insecurity among U.S. college students: a multi-institutional study

**DOI:** 10.1186/s12889-019-6943-6

**Published:** 2019-05-29

**Authors:** Aseel El Zein, Karla P. Shelnutt, Sarah Colby, Melissa J. Vilaro, Wenjun Zhou, Geoffrey Greene, Melissa D. Olfert, Kristin Riggsbee, Jesse Stabile Morrell, Anne E. Mathews

**Affiliations:** 1 Food Science and Human Nutrition Department, University of Florida, PO Box 110370, Gainesville, FL 32611-0370 USA; 2 Department of Family, Youth & Community Sciences, University of Florida, PO Box 110310, Gainesville, FL 32611-0370 USA; 3 Department of Nutrition, University of Tennessee, 229 Jessie Harris Bldg., Knoxville, RN 37996 USA; 4 Department of Business Analytics and Statistics, University of Tennessee, 916 Volunteer Blvd, UT SMC 247, Knoxville, TN 37996 USA; 5 Nutrition and Food Sciences, University of Rhode Island, 125 Fogarty Hall, Kingston, RI 02881 USA; 6 Animal and Nutritional Sciences, West Virginia University, 1194 Evansdale Drive, G28 Ag. Sc. Bldg., Morgantown, WV 26506 USA; 7 Agriculture, Nutrition, and Food Systems, University of New Hampshire, 115 Kendall Hall, 129 Main Street, Durham, NH 03814 USA

**Keywords:** Food insecurity, Food pantry, College students, BMI, Stress, Sleep, Disordered eating, GPA

## Abstract

**Background:**

College students may be vulnerable to food insecurity due to limited financial resources, decreased buying power of federal aid, and rising costs of tuition, housing, and food. This study assessed the prevalence of food insecurity and its sociodemographic, health, academic, and food pantry correlates among first-year college students in the United States.

**Methods:**

A cross-sectional study was conducted among first-year students (*n* = 855) across eight U.S. universities. Food security status was assessed using the U.S. Department of Agriculture Adult Food Security Survey Module. Cohen’s Perceived Stress Scale, Pittsburgh Sleep Quality Index, and Eating Attitudes Test-26 were used to assess perceived stress, sleep quality, and disordered eating behaviors, respectively. Participants self-reported their grade point average (GPA) and completed questions related to meal plan enrollment and utilization of on-campus food pantries.

**Results:**

Of participating students, 19% were food-insecure, and an additional 25.3% were at risk of food insecurity. Students who identified as a racial minority, lived off-campus, received a Pell grant, reported a parental education of high school or less, and did not participate in a meal plan were more likely to be food-insecure. Multivariate logistic regression models adjusted for sociodemographic characteristics and meal plan enrollment indicated that food-insecure students had significantly higher odds of poor sleep quality (OR = 2.32, 95% CI: 1.43–3.76), high stress (OR = 4.65, 95% CI: 2.66–8.11), disordered eating behaviors (OR = 2.49, 95% CI: 1.20–4.90), and a GPA < 3.0 (OR = 1.91, 95% CI: 1.19–3.07) compared to food-secure students. Finally, while half of the students (56.4%) with an on-campus pantry were aware of its existence, only 22.2% of food-insecure students endorsed utilizing the pantry for food acquisition.

**Conclusions:**

Food insecurity among first-year college students is highly prevalent and has implications for academic performance and health outcomes. Higher education institutions should screen for food insecurity and implement policy and programmatic initiatives to promote a healthier college experience. Campus food pantries may be useful as short-term relief; however, its limited use by students suggest the need for additional solutions with a rights-based approach to food insecurity.

**Trial Registration:**

Retrospectively registered on ClinicalTrials.gov, NCT02941497.

## Background

Today nearly 70% of high school graduates directly transition to post-secondary education in pursuit of a college degree [1]. Despite this ostensibly accessible system of higher education, the cost of attending college greatly exceeds the financial means of most students [2]. Major cuts in state support for public colleges has precipitated a rise in the price of attending a public college, a rise that has outpaced growth in median income [2, 3]. Federal support through student aid and tax credits has done little to compensate [2] and, although financing through student loans is nearly ubiquitous, students are not always able to secure adequate support through loans or deliberately choose not to out of fear of accruing excess debt [4]. Thus, transitioning to college might be more difficult than many college students anticipated [5]. The increased financial burden that students encounter may impact their spending priorities. Students often have to prioritize their available budget for rent, tuition, and utilities, while using the remaining insufficient balance for food, which increases their risk of food insecurity [6]. While there is a consensus that pursuing a university degree is an important determinant of social capital and health [7], experiences with food insecurity undermine the socioeconomic agenda of post-secondary education.

Food insecurity is defined as the limited or uncertain access to nutritionally adequate, safe, and acceptable foods that can be obtained in socially acceptable ways [8]. Experiences with food insecurity can refer to running out of food and being unable to afford more; having anxiety about affording meals, or eating a poor-quality diet as a result of limited financial ability [8]. The United States Department of Agriculture (USDA) classifies individuals on a continuum with respect to food security status. Those with *high food security* do not experience any issues stemming from consistent access to adequate food items. *Marginally food-secure* individuals experience anxiety over food sufficiency but are still able to maintain access to desired foods. *Individuals with low food secur*ity experience reduced quality, variety, and desirability of their dietary choices but with little or no indication of a reduction in food intake. Finally, individuals who experience *very low food security* demonstrate multiple indications of disrupted eating patterns and reduced food intake [8].

First-year college students are uniquely susceptible to food insecurity as they are in a period of transition into their new-found autonomy [5], while also learning how to cope with an environment away from home [5]. Many of these students experience considerable difficulty in managing a variety of tasks that they are unaccustomed to, including managing their finances [9]. Added to this challenge is the diminished social support resulting from prolonged emotional and physical separation from their family and friends [10], the effects of which may jeopardize normal eating patterns. First-year college students may also have poor nutrition knowledge, limited earning potential, and lack of budgeting skills and resources required for healthy food preparation [11–13]. Additionally, they may experience higher rates of weight gain and poor eating behaviors, compared to older students [14]. For these reasons, the first year of college has been described as a ‘critical developmental window’ for preventing weight gain, [15] that is paradoxically associated with food insecurity [16].

An increasing number of studies have drawn attention to the high rates of food insecurity experiences on college campuses in the United States [17]. In a recent systematic review [17], the average student food insecurity rate in the U.S. was found to be 32.9% with a range of 14.1% [18] in an urban university in Alabama to 59.0% at a rural university in Oregon [19]. The pervasiveness of campus-based food pantries is also a potential indicator that food insecurity is a salient problem at post-secondary institutions [20]. Across studies, post-secondary students who report food insecurity are more likely to identify as racial minority [21], be financially independent, have an annual income < $15,000, live off-campus with roommates [19], receive a Pell grant [21], be employed while in school [19] and have low self-efficacy for cooking nutritious meals [18] and financial and food literacy skills [18, 22].

Even if student food insecurity is only experienced during the time required to earn a degree, limited access to nutritious foods can precipitate poor health behaviors and increased risks of chronic disease over time. Compared with food-secure students, food insecure students eat fewer fruits, vegetables, and legumes [23], consume more processed meals in order to afford enough food [13], have lower odds of consuming breakfast and home-cooked meals [24] and are less physically active [25]. Consequently, prolonged exposure to food insecurity may contribute to the development of obesity [16] and associated co-morbidities such as hypertension, diabetes, and cardiovascular diseases [26, 27]. Food insecurity also appears to be related to poor mental health and academic performance. Indeed, it has been posited that food insecure students endorse increased rates of depression and anxiety [24, 28], decreased ability to concentrate [29], and low grade point averages compared to their counterparts [29]. Thus, food insecurity can lead to sub-optimal health and lower academic achievement, undermining the goals of tertiary education.

The extent to which first-year college students are at risk of food insecurity remains to be characterized, as research related to food insecurity among this population is currently limited [24, 28, 30]. Unlike the present study that included students from eight geographically diverse institutions and utilized on-site anthropometric and survey assessments, previous studies were limited to small samples from a single institution and reliance on self-reported data collection methods. The present study also provides a rare glimpse of the use and awareness of campus-based food pantries, one of the fastest growing movements to combat food insecurity on university campuses.

To address these gaps in the literature, the present study aimed to: (i) identify and describe the prevalence of food insecurity, (ii) assess the awareness and use of campus food pantries, and (iii) examine the differences in health, academic, and sociodemographic characteristics by food security status of first-year college students from eight U.S. universities. Our overall research question was, *Is food insecurity related to health and academic outcomes in U.S. first-year college students?* We hypothesized that food-insecure students would have poorer mental and physical health outcomes, and lower academic performance compared to food-secure students. Findings from this project will support the development of evidence-based campus initiatives and policies to address student hunger and financial challenges.

## Methods

### Study design

Data were acquired during the project development phase of a USDA-funded, multi-state, prospective health promotion study, *Get FRUVED.* Participants included first-year students (*n* = 855) from eight U.S. universities (University of Florida, University of Maine, University of Tennessee, Auburn University, South Dakota State University, Kansas State University, Syracuse University, and West Virginia University). These universities were members of an established multi-state research team (NC1193). Assessments were conducted at each university during fall 2015 and late spring 2016 academic semesters by trained research assistants. To reflect on food insecurity experienced during the students’ first year of college, data from the second assessment point were utilized for this investigation. The University of Tennessee Institutional Review Board reviewed and provided ethical approval for all study activities at West Virginia University, South Dakota State University, University of Maine, Syracuse University and the University of Tennessee. The Institutional Review Boards at the University of Florida, Auburn University, and Kansas State University reviewed and approved the study for their respective campuses. Participants provided written informed consent prior to completing the assessment procedures.

### Participant recruitment and enrollment

Recruitment of first-year students occurred by campus-wide announcements and advertising through e-mails, orientation events, social media, and campus informational booths. To be eligible, participants had to report eating less than 2 cups of fruits and/or less than 3 cups of vegetables as measured by the National Cancer Institute’s screener [31] and having at least one additional risk factor for weight gain during the college years. The risk factors included any of the following: have a body mass index (BMI) ≥ 25 kg/m^2^, be a first-generation college student, have a parent who is overweight or obese, identify as a racial minority or be of a low-income background [32]. These eligibility criteria were selected in accordance with the objectives of the larger study which was to improve fruit and vegetable intake and other health behaviors among college students. After providing consent, participants completed on-site anthropometric measurements and surveys administered through a secure web-based format.

### Measures

#### Food insecurity

The prevalence of food insecurity over the last 12 months was assessed using the 10-item validated USDA Adult Food Security Survey Module (AFSSM) [33]. The AFSSM measures several conditions and behaviors that are characteristic of food insecurity, including anxiety over food supply, reduced quality and quantity of food consumed, and meal skipping due to lack of financial resources to obtain food. According to the *Guide to Measuring Food Security* [34], the number of affirmative responses was summed to obtain a raw score ranging from 0 to 10. Students were then designated to one of four food security categories: high food security (i.e., no food access problems, defined as having a raw food security score zero), marginal food security (i.e., anxiety over food supply, defined as having a raw food security score 1–2), low food security (i.e., reduced diet quality and variety, defined as having a raw food security score 3–5), or very low food security (i.e., multiple indications of disrupted eating patterns and reduced food intake, defined as having a raw food security score 6–10). For analysis, food security status was dichotomized into *food-secure* (high food security or marginal food security status) and *food-insecure* (low food security or very low food security status) in accordance with the U.S. Department of Agriculture (USDA) Economic Research Service (ERS) [8].

#### Anthropometry

Anthropometric measurements (weight, height, and waist circumference) for study participants were conducted by trained research assistants using a standardized protocol and calibrated equipment. Participants were weighed on a digital scale (Tanita Scale SECA 874) to the nearest 0.1 kg while wearing minimal clothing. Standing height was measured using a portable stadiometer (SECA 213) to the nearest 0.1 cm. BMI was calculated by dividing weight in kilograms by the height in square meters (kg/m^2^). Waist circumference was measured at the midpoint between the lowest palpable rib and the top of the iliac crest and was recorded to the nearest 0.1 cm. Height, weight, and waist circumference measurements were taken twice, and measurements within a pre-specified margin of error were averaged.

#### Sleep quality

Sleep quality was measured using the 19-item Pittsburgh Sleep Quality Index (PSQI) [35], a reliable and valid questionnaire designed to assess sleep quality over the past month [35, 36]. The PSQI yields a total score ranging from 0 to 21 with higher scores indicating worse sleep quality. A total score greater than 5 indicates a “poor” sleeper [35].

#### Perceived stress

Perceived stress was measured using the 14-item Cohen’s Perceived Stress Scale (PSS) [37]. The PSS measures the degree to which situations experienced during the past month are perceived as stressful. Each PSS item yields a score that ranges from 0 to 4, with 4 indicating the highest perception of stress. These item scores were summed to yield a total score ranging from 0 to 56 with higher scores indicating higher stress. Based on previous studies [38, 39], a stress score of 28 or higher was classified as high stress.

#### Disordered eating

Disordered eating behaviors were measured using the Eating Attitudes Test-26 (EAT-26) [40], which assesses symptoms characteristic of eating disorders. Survey items scores were summed for a total score that ranges from 0 to 78. A score of 20 or higher indicates problematic eating behaviors and high risk of disordered eating [41]. The EAT-26 is a reliable and valid instrument that correlates with clinical and psychometric variables [40, 42].

#### Food pantry use and awareness

Students were asked to report whether a campus-based food pantry existed on their campus. Subsequent analysis of the awareness of the food pantry was assessed by calculating the number of students affirming the existence of a food pantry on their campuses when a food pantry was operating at the time of the assessment. For those affirming that their school had a food pantry, they were asked whether they utilize the pantry to obtain food. Finally, the preference for the pantry location was assessed. The three response options included ‘in the center of the campus’, ‘in the center of the campus and hidden’ and ‘on the outskirts of campus with bus access’.

#### Sociodemographic characteristics

Data on participants’ age, sex, race/ethnicity, meal plan, parental education, place of residence, employment, university, and Pell grant status (need-based federal financial aid) were collected. Age was assessed using nine categorical options, which were then grouped into two levels (i.e., 18 years or 19 years and older) due to skewness. Place of residence was assessed with five categorical options, which were then grouped into the ‘On-campus’ and ‘Off-campus’ levels. Participants were asked whether they were enrolled in a meal plan or received a Pell grant with responses available as ‘yes’ or ‘no’. Mother’s and father’s education were assessed using five response options, which were then coded as ‘some college or higher’ and ‘high school or less’. Participants also identified their race using seven response options asking respondents to select all that apply. Another question asked for self-identified ethnicity (i.e., ‘Are you Hispanic or Latino?’) and the available options were ‘yes,’ ‘no,’ and ‘I don’t know/not sure.’ These were then coded as one race and ethnicity variable with four levels: ‘Non-Hispanic white’, ‘Non-Hispanic black’, ‘Hispanic/Latino’, and ‘Other/multi-racial’. Finally, GPA response options included 0.5-point range options from < 2.5 to 3.5–4.0.

### Statistical analysis

Descriptive statistics were used to describe the prevalence of food insecurity and participants’ characteristics. Chi-square test of independence was used to determine the bivariate associations of food insecurity and sociodemographic variables. Whenever the number in any cell was < 5 in a 2 × 2 contingency table, Fisher’s exact test was used. The difference between food-secure and food-insecure students on health-related parameters was analyzed using independent t-test for data that pass the normality test and Mann–Whitney’s U test for those not. To model the association of health and academic outcomes (i.e., BMI, perceived stress, disordered eating behaviors, sleep quality, and self-reported GPA) and food security status, multiple logistic regressions were used. These models were adjusted for variables found to be significant in the bivariate analyses (i.e., Pell grant status, parental education, place of residence, and meal plan status) and variables known to affect outcome measures (age, sex, university, and employment status) based on previous literature [6, 19, 43, 44]. Results from these regression models were reported as odds ratios and 95% confidence intervals. All analyses were conducted using the IBM SPSS Statistics for Windows, version 24 (Armonk, NY). Statistical significance was determined at *P* < 0.05.

## Results

### Participant eligibility and sample size

A total of 5426 students completed eligibility surveys from all eight universities. Of these, 85.3% (*n* = 4630) were enrolled in one of the eight universities and were at least 18 years old. Among the 4630 students, 86.5% (*n* = 4007) had less than optimal fruit and vegetable consumption (< 2 cups of fruit/d and/or < 3 cups of vegetable/d), 24.3% (*n* = 1127) had a BMI ≥ 25 kg/m^2^, 17.6% (*n* = 814) self-identified as first-generation college student, 35.7% (*n* = 1651) had overweight or obese parent, 27.4% (*n* = 1269) self-identified as a racial minority, and 0.8% (*n* = 35) were from low-income background. This criteria resulted in 2757 students eligible to enroll in the study.

Across the eight campuses, 1149 (41.7%) of eligible students chose to enroll in the study and completed a baseline assessment in the fall of 2015. Of these, 860 (74.8%) completed the second assessment during late spring 2016 which was utilized for this investigation. Participants who did not provide a full response to the ten USDA AFSSM questions were excluded from analyses (*n* = 5), leaving data from 855 students as the study sample of this investigation.

### Participant characteristics

Respondents were predominantly female (68.8%), 19 years old (65.4%), and non-Hispanic white (62.4%). Around 43% of the students were employed, and the majority lived on-campus (84.4%) and had a meal plan (80%). The mean BMI was 24.7 ± 5.2 kg/m^2^. Over half of the respondents (58.6%) fell in the normal BMI category (i.e., BMI ranging from 18.5 to 24.9), followed in prevalence by the overweight (i.e., BMI ranging from 25.0 to 29.9) category (25.9%). About 28.5% of the students that were assessed came from the University of Florida, followed by Syracuse University (15.2%), University of Maine (15.0%), Kansas State University (11.0%), University of Tennessee (10.3%), West Virginia University (8.2%), Auburn University (6.5%), and South Dakota State University (5.2%).

Descriptive statistics of the student sample by food security status and associations between food security status and sociodemographic characteristics are presented in Table 1. Using bivariate analysis, food security status was significantly associated with race/ethnicity (*p* < 0.001), Pell grant status (*p* < 0.001), meal plan status (*p* = 0.001), place of residence (*p* = 0.001), and mother’s and father’s education (*p* < 0.001). Specifically, the proportion of students who identified as Black or Hispanic/Latino was greater among food-insecure than food-secure students, and a greater proportion of food-insecure students reported having a parent with a high school degree or less. Findings also indicated that students residing off-campus, receiving a Pell grant, or not enrolled in a meal plan were significantly more likely to be food-insecure than their counterparts. Of note, meal plan enrollment was significantly associated with place of residence (*p* < 0.001). A higher proportion of students participating in a meal plan resided on-campus compared to their counterparts (92.5% versus 7.5%).

**Table 1:** Descriptive characteristics by food security status among first-year college students at risk of weight gain in the United States (*n* = 855), 2016

	All Students (n = 855)^a^	Food-Secure (*n* = 692 [81%])	Food-Insecure (*n* = 163 [19%])	*P*-value^b^ Insecure vs. Secure
Age (y), *n* (%)				0.310
18	293 (34.6)	243 (35.3)	50 (31.2)	
≥ 19	555 (65.4)	445 (64.7)	110 (68.8)	
Sex, *n* (%)				0.391
Male	262 (31.2)	217 (31.8)	45 (28.3)	
Female	579 (68.8)	465 (68.2)	114 (71.7)	
Race/ethnicity, *n* (%)				< 0.001
Non-Hispanic white	434 (62.4)	376 (66.0)	58 (46.0)	
Non-Hispanic black	87 (12.5)	59 (10.4)	28 (22.2)	
Hispanic/Latino	72 (10.3)	51 (8.9)	21 (16.7)	
Other/multi-racial	103 (14.8)	84 (14.7)	19 (15.1)	
Father’s Education Level, *n* (%)				< 0.001
Some college or higher	406 (49.8)	355 (53.1)	51 (34.5)	
High school or less	410 (50.2)	313 (46.9)	97 (65.5)	
Mother’s Education Level, *n* (%)				< 0.001
Some college or higher	469 (56.3)	401 (59.3)	68 (43.3)	
High school or less	364 (43.7)	275 (40.7)	89 (56.7)	
Employment Status, *n* (%)				0.652
Employed (Part-time/full-time)	366 (43.3)	295 (42.9)	71 (44.9)	
Unemployed	479 (56.7)	392 (57.1)	87 (55.1)	
Pell Grant Recipient, *n* (%)				< 0.001
Yes	323 (39.7)	233 (35.4)	90 (58.4)	
No	490 (60.3)	426 (64.6)	64 (41.6)	
Place of Residence, *n* (%)				0.001
On-campus	718 (84.4)	599 (86.3)	119 (75.8)	
Off-campus	133 (15.6)	95 (13.7)	38 (24.2)	
Meal Plan Enrollment, *n* (%)				0.001
Yes	681 (80.0)	568 (82.2)	113 (70.6)	
No	170 (20.0)	123 (17.8)	47 (29.4)	

^a^Counts will not always sum to 855 because of missing data

^b^χ^2^ *P-*values compare the difference by food security status and sociodemographic characteristics; *P*-value < 0.05 is statistically significant

### Prevalence of food insecurity

Responses to the AFSSM indicated that 692 (81.0%) students were food-secure with 476 (55.7%) having high food security and 216 (25.3%) with marginal food security. The remaining 163 (19%) students were classified as food-insecure, consisting of 103 (12.0%) with low food security and 60 (7.0%) with very low food security (Table 2). The highest prevalence of food insecurity (low + very low food security) was observed among students attending the University of Tennessee (25.0%) while the lowest was for West Virginia University (7.1%).

**Table 2:** Prevalence of high, marginal, low, and very low food security among first-year college students at risk of weight gain in the United States (*n* = 855), 2016

University	Total *(n)*	Food-secure *n (%)*			Food-insecure *n (%)*		
		All	High Food Security	Marginal Food Security	All	Low Food Security	Very Low Food Security
Auburn University	56	43 (76.8)	32 (57.1)	11 (19.6)	13 (23.2)	10 (17.9)	3 (5.4)
University of Florida	244	191 (78.3)	129 (52.9)	62 (25.4)	53 (21.7)	34 (13.9)	19 (7.8)
Syracuse University	130	110 (84.6)	83 (63.8)	27 (20.8)	20 (15.4)	13 (10.0)	7 (5.4)
University of Tennessee	88	66 (75.0)	47 (53.4)	19 (21.6)	22 (25.0)	11 (12.5)	11 (12.5)
University of Maine	129	108 (83.7)	75 (58.1)	33 (25.6)	21 (16.3)	14 (10.9)	7 (5.4)
South Dakota State University	44	38 (86.4)	26 (59.1)	12 (27.3)	6 (13.6)	3 (6.8)	3 (6.8)
West Virginia University	70	65 (92.9)	40 (57.1)	25 (35.7)	5 (7.1)	3 (4.3)	2 (2.9)
Kansas State University	94	71 (75.5)	44 (46.8)	27 (28.7)	23 (24.5)	15 (16.0)	8 (8.5)
Total	855	692 (80.9)	476 (55.7)	216 (25.3)	163 (19.1)	103 (12.0)	60 (7.0)

### Health correlates of food insecurity

Significant associations were noted when comparing food-insecure and food-secure students on health variables (Table 3). Accordingly, food-insecure students had significantly higher perceived stress (*p* < 0.001), disordered eating behaviors (*p* = 0.001), and poorer sleep quality compared to food-secure students (*p* < 0.001). There were no significant differences between food-insecure and food-secure students with respect to BMI and waist circumference.

**Table 3:** Health and academic variables by food security status among first-year college students at risk of weight gain in the United States (*n* = 855), 2016

	All Students (*n* = 855)	Food-Secure (*n* = 692 [81%])	Food-Insecure (*n* = 163 [19%])	*P*-value^a^ Insecure vs. Secure
Waist Circumference (cm), Mean ± SD	76.7 ± 5.9	79.1 ± 7.4	79.9 ± 13.2	0.471
BMI (kg/m^2^)				
Mean ± SD	24.70 ± 5.23	24.5 ± 5.0	25.2 ± 5.8	0.112
Overweight/obese (BMI ≥ 25), *n* (%)	310 (37.1)	247 (36.5)	63 (39.9)	0.423
Perceived Stress				
Mean ± SD	27.0 ± 5.9	26.2 ± 5.8	30.2 ± 5.7	< 0.001
High stress^b^, *n* (%)	457 (54.2)	342 (49.8)	114 (73.7)	
Sleep Quality				
Mean ± SD	5.8 ± 2.5	5.4 ± 2.4	6.8 ± 2.8	< 0.001
Poor sleep quality^c^, *n* (%)	542 (64.7)	416 (61.1)	126 (80.3)	< 0.001
Disordered Eating				
Mean ± SD	7.49 ± 7.49	7.0 ± 6.9	9.5 ± 9.1	0.001
Yes^d^, *n* (%)	62 (7.6)	43 (6.5)	19 (12.3)	0.011
GPA, *n* (%)				0.001
3.50–4.00	423 (50.6)	361 (53.3)	62 (38.9)	
3.00–3.49	246 (29.4)	195 (28.9)	51 (32.1)	
2.50–2.99	124 (14.8)	91 (13.4)	33 (20.8)	
< 2.50	43 (5.1)	30 (4.4)	13 (8.2)	

^a^*P*-value < 0.05 is statistically significant

^b^On a scale of 0 to 56, with higher numbers indicating more stress. The score was dichotomized at 28, with scores ≥ 28 considered high stress [37, 38]

^c^On a scale of 0 to 21, with higher numbers indicating worse sleep quality. The score was dichotomized at 5, with scores ≥ 5 considered poor [35]

^d^On a scale of 0 to 78, with higher numbers indicating higher level of problematic eating behaviors and a high level of concern about dieting and body weight. The score was dichotomized at 20, with scores ≥ 20 indicating disordered eating [40, 41]

Multivariate logistic regression analyses controlling for age, sex, race/ethnicity, parental education, meal plan enrollment, employment status, place of residence, and Pell grant status (Table 4) showed that food-insecure students had significantly higher odds of being classified as having high stress (OR = 4.65, 95% CI: 2.66–8.11), disordered eating behaviors (OR = 2.49, 95% CI: 1.20–4.90), and poor sleep quality (OR = 2.32, 95% CI: 1.43–3.70). Association of food insecurity with being overweight was not statistically significant.

**Table 4:** Multivariate logistic regression models examining the association between food insecurity and health and academic outcomes among first-year college students at risk of weight gain in the United States^a^ (*n* = 855), 2016

Dependent Variable	Odds Ratio	95% CI	*P-*value
Overweight/obese (BMI ≥ 25 kg/m^2^)	1.28	0.84 to 1.96	0.242
High Stress^b^	4.65	2.66 to 8.11	< 0.001
Poor Sleep Quality^c^	2.32	1.43 to 3.76	0.001
Disordered Eating^d^	2.49	1.20 to 4.90	0.010
GPA (< 3.0)	1.91	1.19 to 3.07	0.007

^a^Models controlled for age, sex, race/ethnicity, place of residence, meal plan, employment, Pell grant status, university, and parental education

^b^On a scale of 0 to 56, with higher numbers indicating more stress. The score was dichotomized at 28, with scores ≥ 28 considered high stress [37, 38]

^c^On a scale of 0 to 21, with higher numbers indicating worse sleep quality. The score was dichotomized at 5, with scores ≥ 5 considered poor [35]

^d^On a scale of 0 to 78, with higher numbers indicating a higher level of problematic eating behaviors and a high level of concern about dieting and body weight. The score was dichotomized at 20, with scores ≥ 20 indicating disordered eating [40, 41]

### Academic correlates of food insecurity

Findings revealed that food security status was significantly associated with self-reported GPA (*p* = 0.001) (Table 3). A significantly higher proportion of food-secure students had a GPA in the 3.50–4.00 category (53.3% versus 38.9%), while a higher proportion of food-insecure students had a GPA in the 2.50–2.59 and < 2.50 categories compared to food-secure students (20.8% versus 13.4%; 8.2% versus 4.4% respectively) (Table 3). When controlling for sociodemographic characteristics (Table 4), food-insecure students had almost twice the risk of having a GPA < 3.00 compared to food-secure students (OR = 1.91, 95% CI: 1.19–3.07).

### Food pantry use and awareness

To assess the students’ knowledge of the food pantry as a food assistance resource on their campus, analysis of actual versus reported food pantry availability was conducted. Among the eight universities, only three had campus food pantries in operation at the time of the assessment: University of Florida, University of Maine, and Syracuse University. While most University of Florida students were aware of the existing campus food pantry (85.6%, *n* = 209), only a third of students attending Syracuse University (29.5%, *n* = 38) and the University of Maine (28.7%, *n* = 37) reported the existence of an on-campus food pantry.

Utilization of the food pantry was also assessed among students reporting the existence of campus food pantries in these three universities (*n* = 284). Results indicated that only 7.7% utilized the pantry for food acquisition (Table 5).

**Table 5:** Associations between campus food pantry variables and food security status among first-year college students at risk of weight gain in the United States, 2016

	Total *n (%)*	Food-secure *n (%)*	Food-insecure *n (%)*	*P*-value^a^ Insecure vs. secure
Campus Has a Food Pantry	855			0.582
Yes	407 (47.7)	326 (47.2)	81 (49.7)	
No	258 (30.2)	207 (30.3)	51 (31.3)	
Choose not to answer	188 (22.0)	157 (22.8)	31 (19.0)	
Missing	2			
Utilize Food Pantry				< 0.001
Among all respondents^b^	407			
Yes	26 (6.7)	11 (3.5)	15 (20.5)	
No	363 (93.3)	305 (96.5)	58 (79.5)	
Among respondents from campuses with a food pantry	274			< 0.001
Yes	21 (7.7)	9 (4.1)	12 (22.2)	
No	253 (92.3)	211 (95.9)	42 (77.8)	
Food Pantry Location Preference				0.161
In the center of campus	381 (44.6)	316 (45.7)	65 (39.9)	
In the center of campus and hidden	297 (34.7)	228 (32.9)	69 (42.3)	
On the outskirts of campus with bus access	96 (11.2)	80 (11.6)	16 (9.8)	
Choose not to answer	81 (9.5)	68 (9.8)	13 (8.0)	

^*a*^χ^2^ test was used. *P*-value < 0.05 is statistically significant. ^b^Question displayed for students who reported the existence of a campus food pantry

Food pantry utilization was also significantly associated with food security status (*p* < 0.001). While a higher proportion of food-insecure students used the food pantry compared to food-secure students (22.2% versus 4.1%), most food-insecure students (77.8%) did not utilize the pantry for food acquisition. Lastly, most of the students preferred an on-campus and central location for the food pantry but approximately one third (34.7%) preferred a hidden location in the center of the campus.

## Discussion

This survey of 855 first-year students from eight U.S. universities indicated that towards the end of their first year of college, 19% were food-insecure and 7.1% reported severe food insecurity. An additional 25.3% of first-year students experienced anxiety about food shortage. Food-insecure students reported higher perceived stress, a greater prevalence of disordered eating behaviors, and poorer sleep quality compared to food-secure students, a finding that remained significant after controlling for sociodemographic correlates of food insecurity. Food security status was also associated with race/ethnicity, place of residence, Pell grant status, parental education, GPA, meal plan enrollment, and food pantry use.

The prevalence of food insecurity in the current study is markedly lower than prevalence estimates reported in previous studies of college students [19, 24, 28, 45]. Of two studies specific to first-year college students, Bruening et al. [24] found a prevalence of 32% while Darling et al. [28] reported a prevalence of 28%. It is worth noting that, not only are the sample sizes considerably smaller than that of the present study, but each is representative of a single institution. Heterogeneity in food security prevalence at the institutional or regional level may partly explain the discrepancy. Furthermore, the availability and extent of support available to prevent food insecurity among students may widely differ between schools. Another factor may be the influence of self-selection bias. As a sub-study of the larger *Get FRUVED* project, the present investigation was limited to students who volunteered for a multi-year study tied to health and wellness and attended a follow-up at the end of their first year in college.

Findings from this study shed light on the multifaceted impact food insecurity may have on college students’ physical and mental health. Students who experienced food insecurity during their first year of college were four times more likely to have high perceived stress and two times more likely to have poor sleep quality compared to food-secure students. These findings are in line with previous results in the scientific literature. Studies among college students have linked food insecurity to poor mental health and high rates of anxiety [28] and perceived stress [25, 28]. Similarly, in a longitudinal study, Heflin and colleagues [46] reported that food insecurity might be a causal or contributing factor for depression among women. With respect to sleep quality, although the association between food insecurity and sleep has not been examined yet among college students, a study of food insecurity and sleep among men and women reported similar findings [47]. Food-insecure men and women were more likely to report sleep complaints compared to their food-secure counterparts [47]. Thus, students experiencing food insecurity may frequently experience other hardships related to physical and mental health [28].

Food insecurity can further influence the students’ health by eliciting disordered eating behaviors. Consistent with a previous study among first-year college students [28], results from this study suggest that students who have experienced food insecurity had higher odds of disordered eating behaviors than their food-secure counterparts. However, it is worth highlighting the possible overlap between disordered eating indices and compensatory behaviors stemming directly from food insecurity. For example, routine abstinence from eating when hungry could be indicative of disordered eating or simply a food-insecure individual’s coping strategy to prolong food supplies. Other studies have shown that food-insecure individuals adopt a ‘feast or famine’ cycle determined by food availability [48] wherein food intake is intentionally limited as resources diminish followed by overeating when food is more available [49]. Although such behaviors may not represent ‘traditional’ disordered eating, previous work suggests that food insecurity may precipitate binge eating behaviors in children [50]. Regardless of the underlying cause, the increased odds of disordered eating behaviors among food-insecure students indicates heightened eating-related psychological stress and possible deviations from healthy eating patterns. Finally, while no difference was found in BMI by food security status, the observed health risks associated with food insecurity may lead to weight gain and associated co-morbidities over time [51–54].

Our results indicate that the burdens of food insecurity may translate to academic challenges. Food-insecure students were approximately two times more likely to have a GPA  < 3.00 compared to food-secure students. This finding is similar to previous evaluations of GPA among food-insecure college students [29, 45]. Morris et al. [45] noted a significant association between food insecurity and GPA in which students in the highest GPA range (≥ 3.00) were more food-secure than students with lower GPAs. Psychological aspects of food insecurity include fatigue, anxiety, sleep deprivation, and physical weakness [55, 56], which may impair the ability to concentrate during class. Previous work has shown that student energy and ability to concentrate worsens as the food insecurity score increases [57]. Thus, the development of support systems to address food insecurity may be an additional approach for schools interested in enhancing students’ academic experience. Nevertheless, self-reported GPA does not provide the full picture when examining students’ success in college. Future research should consider incorporating additional metrics of academic success such as retention and on-time graduation rates.

This investigation provides insight into the relationship between food security status and students’ characteristics. Significant associations were identified between food insecurity and race/ethnicity, parental education, Pell grant status, place of residence, and meal plan enrollment. Students who identified as Black or Hispanic/Latino and had a low parental education were at increased risk of food insecurity, which is consistent with national data from the general population [41] as well as findings from a large study among college students [45]. Although living off-campus and not being enrolled in a meal plan were each associated with food insecurity, these two variables are highly related as meal plan enrollment is generally required among students residing on-campus but not for those off-campus. This observation is substantiated by a significant association between meal plan enrollment and place of residence among our sample. Access to affordable food off-campus may be more limited than through campus dining halls. Food-insecure students also reported that the lack of reliable transportation hindered food access [6]. Hence, living and eating off-campus may challenge students’ financial management skills more than living on-campus with a meal plan. Collectively, these characteristics can provide a framework for the development of interventions and support systems targeted to those most at risk of food insecurity.

College students who experience financial hardships or inability to afford food may seek aid from a few available resources. The United States Department of Education distributes the Federal Pell grant, a need-based program that is awarded for low-income students for 12 semesters. In the present study, students receiving Pell grant awards were more likely to be food-insecure. The implications of this finding may challenge the adequacy of the buying power of Pell grants currently available for students in financial need. While the cost of tuition reached an average of $9970 in the year of 2017–2018 [58], the maximum Pell grant awarded in the year of 2017–2018 was $5920 [59]. In addition to the Pell grant program, the Supplemental Food Assistance Program (SNAP) provides a safety net for food insecure individuals; however, its eligibility criteria are very restrictive for university students. To be eligible, students must work at least 20 h per week, have dependents and not have child care, and participate in work-study programs. Lastly, meal plan enrollment alone does not appear to promote food security, as approximately 70% of food-insecure students reported having a meal plan. The term ‘meal plan’ traditionally encompasses a range of plans offered by the school, each based on the extent of access provided to the student. While some plans allow for unlimited access throughout the week, others are limited to one meal per day and even no meals on weekends. Clearly these limited plans would not guarantee food security and, the all-you-can-eat policy at most campus dining halls may even perpetuate the feast-famine eating cycle, previously associated with binge eating, and weight gain [50, 54]. Thus, even students who are enrolled in a meal plan or receive federal financial help may still be vulnerable to food insecurity.

In the wake of the cuts in federal and state funding and heightened food insecurity, campus food pantries have been the fastest growing form of emergency relief. Despite the recent increase in the number of food pantries [20], descriptions of students’ use of this resource are limited. In the present study, only 7.7% of the student population utilized the food pantry, a finding that is comparable to our previous results of students at the University of Florida [21]. Many students refuse to use an on-campus food pantry because of the stigma attached to its use or the sense that the food pantry is not intended for them [21], as its need implies a personal failure. Access barriers such as limited hours, regulated frequency of use, and lack of knowledge on the logistics of its use, have also been reported by students [60]. Nonetheless, while the best-funded U.S. approaches to household food insecurity are charitable food-assistance programs, food pantries cannot end hunger or provide a nutritious food supply [61]. Donated food is often not appealing and limited in key nutrients [60]. In fact, food pantry users prefer and need fresh produce, dairy products, eggs, and meat above the canned food provided in the emergency food systems [62]. Collectively, to make the college experience more equitable for students, research and upstream solutions to student food poverty should go beyond the boundaries of need-based food pantries, to a broader food system, with a “rights-based approach to food security” [63].

The results of this study should be interpreted with consideration of its limitations. Sampling bias stemming from the study design may have influenced overall food insecurity prevalence. Thus, it is important to consider when interpreting these findings that the study population is restricted to students who met the eligibility for the *Get FRUVED* project. Nevertheless, although the prevalence of food insecurity may have been lower than other studies of first-year college students [24, 28, 30], the relationship between food insecurity, sociodemographic, health and academic parameters is similar to other reports in the literature [24, 28, 29]. The cross-sectional design of this study only permitted examining associations rather than establishing potential causation between food insecurity and health and academic parameters. Longitudinal and intervention studies that elucidate the mechanisms by which food security can improve health and educational outcomes are needed. Despite the anonymity of the survey, the food security questionnaire items are prone to recall and social desirability biases related to self-report and social stigma associated with food insecurity [21, 64], which may limit the validity of the results. Additionally, food security survey items address questions referencing the past 12-months. Given that data collection occurred at the end of the spring semester (April 2016), a portion of that 12 months window included time prior to students’ enrollment in college. However, consistent with other studies [24, 30] we believe that capturing the experience of first-year college students is of utmost importance, as attending a university is a period where food insecurity may become an issue, for those experiencing financial constraints and social pressures in their new-found autonomy [5]. Finally, although we used USDA AFSSM to assess food insecurity among our sample, the psychometric properties of this survey among college students have not been evaluated.

## Conclusion

This study provides insight into the relatively obscure area of food insecurity among first-year college students and builds upon the scant literature currently available. Findings identify important sociodemographic correlates of food insecurity, affirm observations from single universities about student hunger, and indicate that the prevalence of food insecurity is high. Our data support previous limited evidence that food-insecure students are at increased risk of adverse health and academic outcomes, the effects of which may impact student retention and health behaviors beyond the college years. If this is indeed the case, the impact would not be limited to the individual, presumably carrying over to the school, state, and national level. Our results substantiate the need for screening for food insecurity among college students and the development of evidence-based support modalities to address food insecurity. Both short-term and long-term approaches can provide an untapped opportunity to mitigate the consequences of food insecurity. These may include indexing Pell grants to tuition inflation, expanding work-study opportunities, providing full meal plan subsidies, hosting on-campus farmers' markets, expansion of the Supplemental Nutrition Assistance Program outreach, and providing university support for financial and food literacy training. Finally, this study underscores several areas in need of development to progress food security research among college students. Specifically, future prospective studies should examine the effect of food insecurity on college student retention, graduation, and health outcomes over time. Additionally, with respect to intervention work, future studies should seek to evaluate strategies aimed at addressing student food insecurity. Such progress is essential for accurately depicting the consequences of food insecurity and ultimately going beyond food security to realizing food rights.

## Abbreviations

AFSSM: Adult Food Security Survey Module
BMI: Body mass index
CI: Confidence interval
GPA: Grade point average
OR: Odds ratio
USDA: United States Department of Agriculture
